# Supplementary material for: Promoting participation in physical activity through Snacktivity: A qualitative mixed methods study
Source: PLoS One. 2023 Sep 11;18(9):e0291040. doi: 10.1371/journal.pone.0291040 (PMC10495025; doi:10.1371/journal.pone.0291040)
Supplement: S1 File — (DOCX) [file pone.0291040.s002.docx]

**Snacktivity- Think Aloud Study**

**Semi-structured Patient Interviews**

Participants will be probed to expand on their responses where necessary.

Interviews will be conducted over the telephone.

**Snacktivity**

1. How are you finding Snacktivity overall?
2. What activity snacks are you doing most regularly? Why?
3. Where are you doing your activity snacks?
4. What time of day do you prefer to do your activity snacks?
5. What snacks have you found most enjoyable and why?
6. Are there any snacks which you haven’t enjoyed and why?
7. What snacks have you found most achievable and why?
8. Is there anything that has prevented you from doing Snacktivity?
9. Is there anything which would help you to do more Snacktivity?
10. Is there anything we could do to improve Snacktivity?
11. How are you getting on with SnackApp and the Snacktivity watch?

**Week 2** (in addition to above)

1. Are there any activity snacks that you do more regularly/ have become a part of your routine? Can you tell us which snacks and why you think these have become habits?
2. Have you created any routines for yourself or times of the day in particular where you are taking part in particular activity snacks?
3. How has Snacktivity impacted you on a daily basis? Does it break up the time you may spend at your desk or sitting down? Has this impacted you positively or negatively? Does it disrupt or enhance your day?
