# Supplementary material for: Promoting participation in physical activity through Snacktivity: A qualitative mixed methods study
Source: PLoS One. 2023 Sep 11;18(9):e0291040. doi: 10.1371/journal.pone.0291040 (PMC10495025; doi:10.1371/journal.pone.0291040)
Supplement: S2 File — (DOCX) [file pone.0291040.s003.docx]

Appendix A–Think aloud study themes and sub-themes

| **Think aloud study themes** | Sub themes | Codes | Participants (identified by 4 digit number) who contributed to this code (number of quotes in brackets) | Total number of references recorded |
| --- | --- | --- | --- | --- |
| **The lived experience of participating in Snacktivity™** | Embracing | Embracing statements and statements of intent | 2004 (6), 2006 (9), 2010 (11), 2014 (3), 2021 (1) | 30 |
|  | Location | Location | 2006 (22), 2010 (7), 2014 (2), 2021 (6) | 37 |
|  | Type | Type | 2006 (62), 2010 (36), 2014 (7), 2021 (25) | 130 |
| **The experience and impact of Snacktivity™** | How Snacktivity™ made them feel | Effects of (general) | 2004 (3), 2006 (5), 2010 (6), 2014 (2), 2021 (9) | 25 |
|  |  | Positive statements and noticeable benefits | 2004 (16), 2006 (34), 2010 (7), 2014 (2) | 59 |
| **Motivation for activity** | Barriers and persistence | Motivational thoughts | 2004 (2), 2006 (5), 2010 (12), 2021 (4) | 23 |
|  |  | Evidence of persistence | 2006 (2), 2010 (9), 2014 (4), 2021 (2) | 17 |
|  |  | Limitations and promoting comments | 2004 (8), 2006 (17), 2010 (8), 2014 (7), 2021 (15) | 55 |
|  |  | Demotivational experience and comments | 2004 (1), 2006 (2), 2010 (5), 2014 (1) | 9 |
|  | Habit | Habit and routine | 2006 (4), 2014 (4), 2021 (1) | 9 |
| **Using and responding to Snacktivity™ Technology** | Using Snacktivity technology | Fitbit | 2004 (4), 2006 (1), 2010 (6), 2014 (20), 2021(3) | 34 |
|  |  | SnackApp | 2004 (14), 2006 (5), 2010 (8), 2014 (2), 2021 (18) | 47 |
|  | Responding to Snacktivity™ technology | Fitbit – value judgement | 2004 (1), 2006 (1), 2010 (2), 2014 (2), 2021(2) | 8 |
|  |  | SnackApp – value judgement | 2004 (3),2006 (4), 2021 (8) | 15 |

Appendix B - Semi-structured interview 1: themes and sub themes

| **Semi-structured interview–1^st^ themes** | Sub themes | Codes | Participants (identified by 4 digit number) who contributed to this code (number of quotes in brackets) | | Total number of references recorded |
| --- | --- | --- | --- | --- | --- |
| **The experience of Snacktivity™** | Type and intensity | Type of physical activity | 2004 (3), 2005 (3), 2006 (11), 2008 (2),2010 (9), 2011 (5), 2012 (8), 2014 (15), 2015 (4), 2021 (7), 2023 (5) | 72 | |
|  | Location | Location | 2004 (1), 2005 (1), 2006 (5), 2008 (1), 2010 (1), 2011 (1), 2012 (2), 2014 (5), 2021 (2), 2023 (1) | 20 | |
| **Motivation for activity** | Barriers and limitations | Motivation – positive | 2004 (2), 2006 (2), 2014 (2), 2021 (2), 2023 (6) | | 14 |
|  |  | Habit building | 2006 (1) | | 1 |
|  |  | Recognition of starting point | 2011 (1), 2014 (1) | | 2 |
|  |  | Physical activity persistence | 2011 (1), 2014 (1), 2023 (5) | | 7 |
|  |  | Physical activity intent | 2004 (7), 2006 (1), 2011 (1), 2014 (3) | | 12 |
|  |  | Negative comments | 2006 (2),2008 (2), 2012 (1), 2014 (1), 2015 (1) | | 7 |
|  |  | Self-assessment of ongoing activity and Snacktivity™ behaviour | 2004 (2), 2005 (1), 2011 (1), 2012 (1), 2014 (2), 2015 (2) | | 9 |
|  |  | Non-Snacktivity™ prompts to physical activity | 2004 (3), 2005 (6), 2006 (1), 2012 (5), 2015 (6), 2021 (4) | | 25 |
|  |  | Barriers and limitations | 2004 (11), 2005 (4), 2006 (10), 2008 (5), 2010 (2), 2011 (14), 2012 (12), 2014 (18), 2015 (4) ,2021 (4), 2023 (4) | | 88 |
| **Experiences of the Snacktivity™ Technology** | Notifications, alerts and prompts | Snacktivity™ prompts activity | 2006 (8), 2008 (2), 2010 (3), 2011 (3), 2012 (3), 2014 (5), 2021 (8), 2023 (2) | | 34 |
|  |  | Reflections upon prompts | 2006 (5), 2008 (1),2010 (2) | | 8 |
|  | Fitbit | Fitbit | 2004 (5), 2006 (2), 2008 (1), 2011 (1), 2012 (2), 2014 (3), 2015 (1),2021(10) | | 25 |
|  | SnackApp | SnackApp | 2004 (3), 2005 (1), 2006 (2), 2008 (3), 2010 (1), 2011 (3), 2012 (2), 2014 (2), 2021(1) | 18 | |
|  |  | Suggested improvements | 2010 (3), 2011 (7), 2012 (4), 2021(5) | 19 | |
|  |  | Lack of understanding of the App | 2006 (4), 2012 (3), 2015 (3), 2021 (9), 2023 (2) | 21 | |
|  |  | Negative comments about the App | 2004 (8), 2005 (3), 2006 (2), 2008 (7), 2010 (3), 2011 (1), 2012 (3), 2014 (4), 2015 (1), 2021 (15), 2023 (2) | 49 | |

Appendix C – Semi-structured interview 2: themes and sub themes

| **Semi-structured interview–2nd themes** | Sub themes | Codes | Participants (identified by 4 digit number) who contributed to this code (number of quotes in brackets) | Total number of references recorded |
| --- | --- | --- | --- | --- |
| **The experience of Snacktivity™** | Location | Home gym | 2006 (1), 2014 (1), 2021 (3), 2023 (3) | 8 |
|  |  | Snack location | 2004 (1), 2005 (1),2006 (2), 2014 (3), 2023 (1) | 8 |
|  | Variety of activity | Type of activity | 2004 (7), 2005 (6), 2006 (6), 2010 (5), 2011 (2), 2012 (1), 2014 (6), 2021 (3), 2023 (6) | 42 |
|  |  | Reason for choice of snacks | 2010 (3), 2011 (1), 2014 (2), 2021(1) | 7 |
|  |  | Limitations upon snacks | 2005 (3), 2006 (3), 2010 (7), 2011 (3), 2012 (2), 2014 (7), 2015 (2), 2021 (2), 2023 (4) | 33 |
|  | Benefit | Benefits expressed | 2006 (5), 2014 (2), 2023 (3) | 10 |
| **Motivation for activity** | Motivation for activity (general) | Positive motivational | 2004 (1), 2005 (2), 2006 (8), 2010 (1), 2012 (1), 2015 (5), 2023 (3) | 21 |
|  |  | Assessment of activity | 2004 (2), 2005 (2), 2006 (1) | 5 |
|  |  | Reappraisal of activity | 2006 (2), 2010 (1), 2012 (1), 2023 (1) | 5 |
|  | Barriers and limitations | Improvement (general) | 2010 (1), 2011 (4), 2012 (2), 2014 (5), 2023 (8) | 20 |
|  |  | Snacktivity™ improvements | 2012 (16), 2015 (1), 2021 (1) | 18 |
|  | Demotivation | Low activity | 2004 (1), 2012 (1) | 2 |
|  |  | SnackApp demotivation | 2004 (5), 2010 (3), 2012 (6), 2014 (3), 2021 (1), 2023 (1) | 19 |
|  | Routine | Establishing routine | 2004 (5), 2005 (4), 2006 (6) 2010 (2), 2011 (1) 2014 (8), 2021 (1), 2023 (3) | 30 |
| **Awareness and impact of being a participant in a health study** |  | Study as motivation | 2004 (5), 2005 (1), 2006 (1), 2010 (5), 2011 (2), 2012 (8), 2014 (1), 2015 (7), 2023 (1) | 31 |
| **Experiences of Snacktivity™ Technology** | Experiences of Snacktivity™ Technology | Device working properly | 2005 (1), 2010 (4) | 5 |
|  | Prompts, notification and alerts | Notification motivation | 2005 (1), 2006 (1), 2010 (2), 2011 (3), 2012 (1), 2014 (1), 2021 (3), 2023 (5) | 17 |
|  |  | More snacks and prompting | 2004 (3), 2006 (7), 2011 (1), 2021 (6), 2023 (1) | 18 |
|  | Fitbit | Fitbit general and positive | 2004 (2), 2005 (3), 2006 (3), 2014 (2), 2015 (1), 2021(3) | 14 |
|  |  | Fitbit negative | 2004 (3), 2005 (2), 2011 (3), 2014 (2), 2015 (1), 2021 (5), 2023 (1) | 17 |
|  |  | Fitbit improvements | 2010 (1) | 1 |
|  | SnackApp | Problems | 2004 (3), 2005 (1), 2006 (3), 2012 (9), 2021 (1) | 17 |
|  |  | Familiarisation | 2006 (2), 2012 (2) | 4 |
|  |  | App use | 2021 (1), 2023 (1) | 2 |
|  |  | Forum use | 2021 (3), 2023 (2) | 5 |
|  |  | Suggested improvements | 2004 (3), 2005 (7), 2006 (2), 2012 (5), 2014 (2) | 19 |

Appendix D: Table demonstrating how analyses (think aloud study, 1^st^, & 2^nd^ semi-structured interviews) were combined into a single set of subthemes and themes

| **Think aloud themes** | Think aloud – Sub themes^a^ | **Semi-structured interview–1^st^ themes** | Semi-structured interview–1^st^ sub themes^a^ | **Semi-structured interview–2nd themes** | Semi-structured interview–2nd sub themes^a^ | **Combined themes** | Combined sub themes^a^ |
| --- | --- | --- | --- | --- | --- | --- | --- |
| **The lived experience of participating in Snacktivity™** | Embracing (30) -  2004 (6), 2006 (9), 2010 (11), 2014 (3), 2021 (1) | **The experience of Snacktivity™** |  | **The experience of Snacktivity™** |  | **The lived experience of participating in Snacktivity™** | Embracing (30) -  2004 (6), 2006 (9), 2010 (11), 2014 (3), 2021 (1) |
|  | Location (37) -  2006 (22), 2010 (7), 2014 (2), 2021 (6) |  | Location (20) -  2004 (1), 2005 (1), 2006 (5), 2008 (1), 2010 (1), 2011 (1), 2012 (2), 2014 (5), 2021 (2), 2023 (1) |  | Location (16) -  2004 (1), 2005 (1), 2006 (3), 2014 (4), 2021 (3), 2023 (4) |  | Location (73) –  2004 (2), 2005 (2),  2006 (30), 2008 (1),  2010 (8), 2012 (2),  2014 (11), 2021 (11), 2023 (5) |
|  | Type (130) -2006 (62), 2010 (36), 2014 (7), 2021 (25) |  | Type and intensity (72) -  2004 (3), 2005 (3), 2006 (11), 2008 (2),2010 (9), 2011 (5), 2012 (8), 2014 (15), 2015 (4), 2021 (7), 2023 (5) |  | Variety of activity (82) –  2004 (7), 2005 (9), 2006 (9), 2010 (15), 2011 (6), 2012 (3), 2014 (15), 2015 (2), 2021 (6), 2023 (10) |  | Type, variety and intensity (284)-  2004 (10), 2005 (12), 2006 (82), 2010 (60), 2011 (11), 2012 (11), 2014 (37), 2015 (6), 2021 (38), 2023 (15) |
|  |  |  |  |  | Benefit (10) –  2006 (5), 2014 (2), 2023 (3) |  | Experience and impact (94) –  2004 (19), 2006 (44), 2010 (13), 2014 (6), 2021 (9),  2023 (3) |
| **The experience and impact of Snacktivity™** | How Snacktivity™ made them feel (84) - 2004 (19), 2006 (39), 2010 (13), 2014 (4), 2021 (9) |  |  |  |  |  |  |
| **Motivation for activity** | Barriers and persistence (104) –  2004 (11), 2006 (26), 2010 (34) 2014 (12), 2021 (21) | **Motivation for activity** | Barriers and limitations (102) -  2004 (25), 2005 (4), 2006 (10), 2008 (5), 2010 (2), 2011 (14), 2012 (12), 2014 (18), 2015 (4) ,2021 (4), 2023 (4) | **Motivation for activity** | Barriers and limitations (38) -  2010 (1), 2011 (4), 2012 (18), 2014 (5), 2015 (1), 2021 (1), 2023 (8) | **Motivation for Snacktivity™** | Barriers and demotivation (244) -  2004 (36), 2005 (4), 2006 (36), 2008 (5), 2010 (37), 2011 (18), 2012 (30), 2014 (31), 2015 (5), 2021 (26), 2023 (12) |
|  |  |  |  |  | Demotivation (21) -2004 (6), 2010 (3), 2012 (7), 2014 (3), 2021 (1), 2023 (1) |  | Routine and habit formation (60) –  2004 (11), 2005 (4), 2006 (10) 2010 (5), 2011 (1) 2014 (15), 2021 (3), 2023 (4) |
|  | Habit (9) -  2006 (4), 2014 (4), 2021 (1) |  |  |  | Routine (30) –  2004 (5), 2005 (4), 2006 (6) 2010 (2), 2011 (1) 2014 (8), 2021 (1), 2023 (3) |  |  |
|  |  |  |  | **Awareness and impact of being a participant in a health study** |  |  | Study driven motivation (not reported) |
| **Using and responding to Snacktivity™ Technology** | Using Snacktivity™ technology (23) –  2004 (4), 2006 (5), 2010 (2), 2014 (2), 2021(10) | **Experiences of the Snacktivity™ Technology** | Notifications, alerts and prompts (42) –  2006 (13), 2008 (3), 2010 (5), 2011 (3), 2012 (3), 2014 (5), 2021 (8), 2023 (2) | **Experiences of Snacktivity™ Technology** | Prompts, notification and alerts (35) –  2004 (3),  2005 (1), 2006 (8), 2010 (2), 2011 (4), 2012 (1), 2014 (1), 2021 (9), 2023 (6) | **Experiences with Technology Snacktivity™** | Fitbit and SnackApp (100) –  2004 (7),  2005 (1), 2006 (26), 2010 (9), 2011 (7), 2012 (4), 2014 (18), 2021 (27), 2023 (8) |
|  | Responding to Snacktivity™ technology (81) –  2004 (18), 2006 (6), 2010 (14), 2014 (22), 2021(21) |  | Fitbit (25) –  2004 (5), 2006 (2), 2008 (1), 2011 (1), 2012 (2), 2014 (3), 2015 (1),2021(10) |  | Fitbit (32) –  2004 (5), 2005 (5), 2006 (3), 2010 (1), 2011 (3), 2014 (4), 2015 (2), 2021 (8), 2023 (1) |  | Notification, alerts and prompts (292) –  2004 (45), 2005 (17), 2006 (26), 2010 (22), 2011 (15), 2012 (28), 2014 (37), 2015 (7), 2021 (74), 2023 (8) |
|  |  |  | SnackApp (107) -  2004 (11), 2005 (4), 2006 (8), 2008 (10), 2010 (7), 2011 (11), 2012 (12), 2014 (6), 2015 (4), 2021 (30), 2023 (4) |  | SnackApp (47) –  2004 (6), 2005 (8),  2006 (7), 2012 (16), 2014 (2), 2021 (5), 2023 (3) |  |  |

^a=^ the code label is given with the number of statements made displayed in brackets, followed by the participant identification number (4 digits) of those contributing data to the code, with the number of statements recorded for each participant for a theme displayed in brackets thereafter.
