# Supplementary material for: Promoting participation in physical activity through Snacktivity: A qualitative mixed methods study
Source: PLoS One. 2023 Sep 11;18(9):e0291040. doi: 10.1371/journal.pone.0291040 (PMC10495025; doi:10.1371/journal.pone.0291040)
Supplement: Attachment — Submitted filename: Plos One responses to reviewers 230723.docx [file pone.0291040.s004.docx]

**PONE-D-23-08763: Promoting participation in physical activity through Snacktivity™: A qualitative mixed methods study**

**Editor requested edits**

Abstract

Line 56-59 this should be part of the methodology and not the background of the abstract.

**RESPONSE: This has been amended.**

Line 130-131: “Participants were recruited to take part in a study that involved both qualitative and quantitative data collection. The report focuses on the qualitative data only. As part of the quantitative data collection procedures, participants completed assessments of health outcomes at the start and end of a three-week Snacktivity™ intervention period (data not reported here).” Why was the quantitative data not reported alongside the qualitative study? This could have expressed the quality of the study giving more explanation based on the findings which will be more appreciated with regards to the same topic. I suggest you include the quantitative aspect of the study which will be better or entirely remove the methodological aspect of the quantitative study in this report.

**RESPONSE: Reference to the quantitative component of the study has been removed.**

Full what is meaning of AD in the study? Please clearly state before using it in the manuscript.

**RESPONSE: AD refers to the author Amanda Daley.**

Recruitment and data: Line 233-236 This part of the report should be under materials and methods

**RESPONSE: This has been changed.**

“A total of 583 invitations were sent to patients using an NHS podiatry service and 47 sent to general practice patients who had participated in an earlier Snacktivity™ study. From these invitations, 20 eligible individuals agreed to take part (6 men & 14 women) in the larger study. No data was gathered regarding reasons for non-participation”. The author can start the sentence with “eleven out of the 20 participants…….”

**RESPONSE: This has been corrected (Lines 234-240).**

Line 261 (Table 2). Table 2 – Themes and subthemes across all three analysis and combined themes. For clarity of the Table, I request that each activity (the 3 interviews and combined data table) has a Table, the themes generated, the subthemes, generated codes and repetition of code by each participant for elaboration on the data analysis conducted.

**RESPONSE: To prevent an excess of tables in the main text, the tables of themes generated for each of the studies/datasets has been included and presented in a supplementary file.**

The translation is not clear, the authors may need to do some punctuations and the grammar to ensure that the person reading it understands. Eg line 270-274. This should be done where required in the whole manuscript.  “You know people always think, you know, you always think, oh you know you need to go and do a twenty minute workout, and I think you know what you’ve shown us is that we can you know choose to do whatever, whatever it is that you enjoy doing, just … just have that moment for two minutes, you know, it doesn’t even have to be five ...”(int2-2006)

**RESPONSE: We have made some minor changes to punctuations and grammar where we can but we wanted to highlight that we are reporting direct speech so it is not always possible to change the text without misreporting what has been said by participants. As is usual practice, occasionally with have used the text (…….) to denote where irrelevant text has been omitted.**

The discussion should not have headings rather, you can use a paragraph for each point that was raised in the discussion. Furthermore, you can discuss the strength of the study under a paragraph while the limitations of the study should be the last paragraph and stand alone. Please ensure that your decision is justified on PLOS ONE’s [publication criteria](https://journals.plos.org/plosone/s/criteria-for-publication) and not, for example, on novelty or perceived impact.
**RESPONSE: The discussion has been edited as requested.**

**Reviewer #1**: This was a very well-written and interesting paper, and this type of rich user experience data for a possible intervention is extremely important and valuable. I have a few minor comments.
**RESPONSE: We would like to thank the reviewer for their kind words.**

I would have liked to see the interview schedule(s) included as supplementary material

**RESPONSE: The interview schedule has been added as supplementary file.**

A little more context in the methods about the two sets of qualitative interviews would be useful (e.g. the process and the rationale for considering these are separate and the decision around timing of these). My initial reading from the methods made me think they were repeat interviews with the same participants, but the results suggested they were different participants. Clarity around this would be helpful.

**RESPONSE: The participants in interviews 1 & 2 were the same people to capture change over time, with the exception of S2008, who was unable to conduct the second interview. We have clarified this on line 194 and in the abstract.**

I wondered if it the table could be reformatted slightly. I realise that is difficult when presenting multiple results, but I didn't not find it very easy to read in its current format. Perhaps (e.g.) a standalone table with the condensed themes might be useful.

**RESPONSE: We have amendment the theme table (Table 2) in the main text and we hope this is clearer now.**

**Reviewer #2**

The Snacktivity approach seems remarkable and like it has great promise! The think aloud component in particular is a strength of this study. I’d like to encourage the authors to cite some more recent literature, and to go a little more in-depth in some areas. As a whole though, this is a strong paper. I have included comments below that should help strengthen this paper.

**RESPONSE: We would like to thank the reviewer for their kind words and feedback.**

Abstract:
How were eleven adults interviewed twice to equal 21?
**RESPONSE: One participants did not complete a second interview. This has been clarified in the abstract and main text (Line 71 and Line 194).**

I’d argue that they weren’t “prevented from” exercising in front of others but chose not to.
**RESPONSE: We appreciate this feedback. We have tried to present findings as expressed by participants and have added the word “perceived” to help clarify this statement**

Acknowledging the timeframe of the study would be useful; pre-COVID-19, doing activity at home was not as accessible as during the timeframe of 2020-December 2021 when working from home was more common. This should also be recognized in presenting the results (e.g., relevant context).
**RESPONSE: Due to the abstract word limit we are not able to add any further information. However, the methods and discussion acknowledge that the study too place during COVID-19.**

Literature Review:
Line 104: while it may be a (perceived) lack of time for some, it is truly a lack of time for others. Perhaps “often” could be added to the parentheses to avoid assigning blame.
**RESPONSE: This line has been edited as suggested by the reviewer-thank you (Line 105).**

Line 105: many of the citations are dated 2007/2010; anything more recent? There have been many more increased demands on people’s times over the past 15 years, particularly given the numerous recessions and economical disasters (plus things like COVID-19).

**RESPONSE: Some references have not been updated as they are seminal papers. However, two references have been updated. (references 7 & 9).**

Methods:
The title leads me to a bit of confusion – how is it both qualitative and mixed-methods? Wouldn’t it just be mixed-methods? But, since only the qualitative portions described here, it should be qualitative right?
**RESPONSE: Yes we believe the title is correct as we have mixed methods by way of the inclusion of the interview study and think aloud study.**

Why did respondents have to be “inactive or moderately inactive” vs already active? It seems that the goal was to increase physical activity, specifically using Snacktivity, so could someone who was already active continue to add some habit-forming behaviors and Snacktivity use in too?

**RESPONSE: We agree that Snacktivity™ may be useful for all citizens regardless of their physical activity but we believe those who are inactive are likely to have the most to gain. For this reason we focused on citizens who were the most inactive.**

I’m curious why podiatry was the specialty that was focused on. Can the authors explain this further?
**RESPONSE: A community health trust is supporting the trial which has a large and research ready podiatry service and therefore we decided to use this service for this study.**

The interviews are a bit on the shorter side. Do the authors still feel like they got everything they could out of the participants related to the topics of interest?
**RESPONSE: A policy of allowing the interviews to be succinct was followed to avoid overburdening participants and thus reducing the likelihood of them declining to participate in the second interview (Lines 198-200). Some participants were also involved in the think aloud study. Whilst the interview were succinct they nevertheless still generated a substantial amount of data for this study, coupled with the think aloud data.**

What was the timeline of the total intervention to completion of the second interview? Could there be a recall bias at all (it doesn’t sound like it from the manuscript as written); just checking.

**RESPONSE: Interviews were conducted at the start and end of the three week Snacktivity™ intervention period (Lines193-195).**

Results: It’s interesting that the second interview study was where “awareness and impact of being a participant in a health study” really emerged for the first time. And why was it combined into the larger set of three themes? It seems pretty distinctive to me. Combining the others makes sense; this is the one I think deserves some attention on its own.
**RESPONSE: Thank you for this feedback. We have commented on the fourth theme in the manuscript (please see lines 250-259). In summary here, the theme ‘awareness and impact of being a participant in a health study’, was not included in the combined themes as it was considered to be primarily of interest to the research team in the methodological design of future Snacktivity™ trials. Information regarding this fourth theme can be found in a supplementary file named ‘Theme coding process’ in Appendix C.**

Why is “stair climbing” capitalized in Table 3?

**RESPONSE: Thank you for highlighting this which has been corrected.**

Going back to the “feeling like they couldn’t snack publicly” – what types of employment did the participants have? I know many people who take video calls while on the treadmill or bike at home or in the office so this is a surprising finding.

**RESPONSE: We do not know the exact professions of participants only what their status was as described in Table 1a.**

It’s interesting that children are an excuse to NOT complete the snacks. Children, especially small ones, should likely be the ones to encourage engagement in jumping and moving! I have young children myself and I find that I am the only one who limits myself with working out around them for short bursts; they want to participate! There may not be anything to add here for the paper but just a thought.

**RESPONSE: Thank you for this comment.**

What does the participant mean, the “trauma” of setting up an app? How is setting up an app traumatic? Now, perhaps this is due to the age of the participant? I’m interested in knowing more.

**RESPONSE: Some of the participants were older adults and as the reviewer highlights some initially had difficulty setting this up. We discuss this point on Lines 522-524.**

Discussion: It is mentioned early on the self-regulation theory and the habit formation model provided the basis for the intervention. Yet, it is not discussed in relationship with the interview or think-aloud findings, nor the implications related. Please expand.

**RESPONSE: We have a paragraph regarding habit formation in the discussion (Lines 510-517). We had discussed the results in the context of self regulation theory but we have now tried to make this more explicit within the discussion.**

Lines 453-454: In what ways were the findings not universal? Any common factors between the individuals?

**RESPONSE: We have added some text to this comment to make it clearer (Lines 454-456.)**

**Journal formatting corrections**

Thank you for stating the following in the Acknowledgments Section of your manuscript: The Snacktivity™ trial is funded by the NIHR (Programme Grants for Applied Research, reference number: RP-PG-0618–20008). AJD is supported by a National Institute for Health Research (NIHR) Research Professorship award. KJ and SG are part-funded by NIHR Applied Research Collaboration (ARC) West Midlands. This research was supported by the NIHR Leicester Biomedical Research Centre. This publication presents independent research funded by the NIHR. The views expressed are those of the author(s) and not necessarily those of the NHS, the NIHR or the Department of Health and Social Care. However, funding information should not appear in the Acknowledgments section or other areas of your manuscript. We will only publish funding information present in the Funding Statement section of the online submission form. Please remove any funding-related text from the manuscript and let us know how you would like to update your Funding Statement. Currently, your Funding Statement reads as follows:

This work was supported by the National Institute for Health Research (NIHR,[www.nihr.ac.uk](http://www.nihr.ac.uk).) (grant reference RP-PG-0618-20008). AJD is supported by a National Institute for Health Research (NIHR, [www.nihr.ac.uk](http://www.nihr.ac.uk).) Research Professorship award. The funders had no role in study design, data collection and analysis, decision to publish, or preparation of the manuscript. Please include your amended statements within your cover letter; we will change the online submission form on your behalf.

**RESPONSE: We have included an amended statement of funding in the cover letter.**

In your Data Availability statement, you have not specified where the minimal data set underlying the results described in your manuscript can be found. PLOS defines a study's minimal data set as the underlying data used to reach the conclusions drawn in the manuscript and any additional data required to replicate the reported study findings in their entirety. All PLOS journals require that the minimal data set be made fully available. "Upon re-submitting your revised manuscript, please upload your study’s minimal underlying data set as either Supporting Information files or to a stable, public repository and include the relevant URLs, DOIs, or accession numbers within your revised cover letter. We will update your Data Availability statement to reflect the information you provide in your cover letter.

**RESPONSE: We have now added the data to the Loughborough University data depository** ['Snacktivity' to promote physical activity and reduce future risk of disease in the population (figshare.com)](https://figshare.com/s/0eea6f01a8f340abbe64). We have also included the data availability statement in the cover letter as requested.

Please amend the manuscript submission data (via Edit Submission) to include Sheila M Greenfield.

**RESPONSE: This has been corrected.**

Please include captions for your Supporting Information files at the end of your manuscript, and update any in-text citations to match accordingly. Please see our Supporting Information guidelines for more information: <http://journals.plos.org/plosone/s/supporting-information>.
**RESPONSE: Captions for supporting information files have been added at the end of the paper.**

While revising your submission, please upload your figure files to the Preflight Analysis and Conversion Engine (PACE) digital diagnostic tool, <https://pacev2.apexcovantage.com/>. PACE helps ensure that figures meet PLOS requirements. To use PACE, you must first register as a user. Registration is free. Then, login and navigate to the UPLOAD tab, where you will find detailed instructions on how to use the tool. If you encounter any issues or have any questions when using PACE, please email PLOS at [figures@plos.org](mailto:figures@plos.org). Please note that Supporting Information files do not need this step.

**RESPONSE: N/A**
