# Supplementary material for: Promoting participation in physical activity through Snacktivity: A qualitative mixed methods study
Source: PLoS One. 2023 Sep 11;18(9):e0291040. doi: 10.1371/journal.pone.0291040 (PMC10495025; doi:10.1371/journal.pone.0291040)
Supplement: Attachment — Submitted filename: PLOS one second set of reviewer responses.docx [file pone.0291040.s005.docx]

**The authors have responded well to the questions but still need to do so adjustments to the manuscript as stated below:**

RESPONSE: We are pleased that we have responded well to the questions raised.

**Line 261 (Table 2). Table 2 – Themes and subthemes across all three analysis and combined themes. For clarity of the Table, I request that each activity (the 3 interviews and combined data table) has a Table, the themes generated, the subthemes, generated codes and repetition of code by each participant for elaboration on the data analysis conducted. The response by the authors with regard to the above question is not satisfactory. The authors need to include the codes for each subtheme generated if the codes were used in generating the subthemes before the formation of the themes. That will not in any way congest the table but will make the table more explanatory with regards to the study. Codes are the basis for a qualitative study. Definitions of terms for the table can be done as foot notes of the table.**

RESPONSE**:** We have provided codes, sub themes and main themes in Table 2 (lines 265-266) in the manuscript and directed readers to supplementary file 4 for further information on theme generation according to each data set and the combined data set used in the study (see Lines 254-255). Additionally, within supplementary file 4 we have provided information for all three individual data sets and the combined themes regarding; main themes, sub themes, codes, ID for participants who contributed to every code and the number of references for a code that were recorded. We believe that collectively this information provides a substantial amount of transparency to readers regarding the process by which the main themes were generated. We believe this is the best approach to provide readers with the information required to assess the methods and findings of the manuscript, whilst keeping the information in the manuscript manageable for readers to digest.

**Kindly state the inclusion and exclusion criteria of the study. The authors stated the eligibility for participation which I assume was the inclusion criteria but what about the exclusion criteria of the study?**

RESPONSE: Both inclusion and exclusion criteria are included now (see Lines 153-156).

**Did you reach ‘data saturation point’ while conducting the interviews? If yes, kindly state it in your methods and if you have not reached “data saturation point” clearly state that as part of the limitation of the study in your discussion. This is relevant in any qualitative study, it hints on the fact whether enough information has been gathered during the study or there is more information that could be explored in the area of the study which has not been harnessed**
RESPONSE: We addressed the issue of saturation in the previous submission but we have added some additional text to the methods and discussion sections (Lines 156-159 and 546-549).
